# *In Vivo* RNAi-Based Screens: Studies in Model Organisms

**DOI:** 10.3390/genes4040646

**Published:** 2013-11-25

**Authors:** Miki Yamamoto-Hino, Satoshi Goto

**Affiliations:** Department of Life Science, Rikkyo University, 3-34-1 Nishi-Ikebukuro, Toshima-ku, Tokyo 171-8501, Japan; E-Mail: hinomiki@rikkyo.ac.jp

**Keywords:** *Drosophila*, genome-wide screen, RNAi library, false results, interaction network

## Abstract

RNA interference (RNAi) is a technique widely used for gene silencing in organisms and cultured cells, and depends on sequence homology between double-stranded RNA (dsRNA) and target mRNA molecules. Numerous cell-based genome-wide screens have successfully identified novel genes involved in various biological processes, including signal transduction, cell viability/death, and cell morphology. However, cell-based screens cannot address cellular processes such as development, behavior, and immunity. *Drosophila* and *Caenorhabditis elegans* are two model organisms whose whole bodies and individual body parts have been subjected to RNAi-based genome-wide screening. Moreover, *Drosophila* RNAi allows the manipulation of gene function in a spatiotemporal manner when it is implemented using the Gal4/UAS system. Using this inducible RNAi technique, various large-scale screens have been performed in *Drosophila*, demonstrating that the method is straightforward and valuable. However, accumulated results reveal that the results of RNAi-based screens have relatively high levels of error, such as false positives and negatives. Here, we review *in vivo* RNAi screens in *Drosophila* and the methods that could be used to remove ambiguity from screening results.

## 1. Introduction

The classical forward genetic approach is a powerful method for the elucidation of genetic and molecular mechanisms. However, this approach is limited to organisms with large numbers of progeny, such as *Escherichia coli*, *Saccharomyces cerevisiae*, *Schizosaccharomyces pombe*, *Caenorhabditis elegans*, *Danio rerio*, and *Drosophila melanogaster*. Even in organisms in which forward genetics is possible, it is essential to develop straightforward and efficient screening methods to perform genome-wide screens, for example, saturation mutagenesis.

Recent breakthroughs, including the development of RNA interference (RNAi) [1] and the determination of whole genome sequences [2,3,4,5], have enabled us to perform genome-wide screens in various organisms. RNAi silencing of a specific target gene relies on the ability of small interfering RNAs (siRNAs), long double-stranded RNAs (dsRNAs), or short hairpin RNAs (shRNAs) to target mRNA molecules for degradation [6,7,8]. This technique has been widely and intensively applied in gene silencing experiments.

The advent of whole genome sequencing opened a new era in the field of biology. Complete genome sequences provide information about the organization and transcribed sequences of all genes in a genome. Based on this information, dsRNAs can be designed to efficiently and specifically reduce the expression of targeted genes. Once dsRNAs covering a whole set of genes have been synthesized, genome-wide screens equivalent to saturation mutagenesis can be implemented.

However, RNAi has two major problems; the delivery and/or expression of dsRNAs in target cells and erroneous results, such as false positives and negatives.

The mode of dsRNA delivery and expression in target cells is dependent on which cells or organisms are used. Mammalian and *Drosophila* cultured cells and model organisms, such as *C. elegans* and *Drosophila*, are intensively used in genome-wide screens. Delivery of dsRNAs into mammalian [9] and *Drosophila* cultured cells [10,11] and *C. elegans* [12,13,14,15] is relatively simple, but expression in the *Drosophila* organism is a labor- and time-intensive process, as injection of dsRNAs into embryos [16,17] or generation of RNAi inducible *Drosophila* strains [18,19,20,21] is required. To avoid the lengthy process of strain generation, three independent libraries of RNAi strains are either currently being, or have already been, generated and are commonly available [22,23,24]. The crossing of library strains with Gal4 driver strains [25] produces knockdown of specific genes in the resulting offspring.

False positives and negatives compromise RNAi-based screen results. In many instances, RNAi-based screens have produced results that were inconsistent with those of separate genome-wide screens, and false results were found to be the main cause of discrepancies between the results obtained by the two methods. False positives, where genes are unexpectedly silenced by dsRNAs, are caused by off-target effects (OTEs) and indirect effects by knockdown of general machineries, for example, general transcription machinery, whereas false negatives are mainly caused by the low silencing efficacies of specific dsRNA molecules. Several disambiguation methods have been proposed, and some have been implemented, for RNAi-based screens, which are described in detail later (Section 7).

In this review, we focus mainly on organism-based (hereafter referred to as *in vivo*) RNAi screens and experimental and computational disambiguation methods that could be used for check the results of these screens.

## 2. RNA Interference

Efficient silencing of gene expression by dsRNA was first discovered by Fire and Mello [1]. Subsequent studies revealed that injected or expressed long dsRNAs are fragmented into ~21 bp small interfering RNA molecules (siRNAs) by Dicer [26,27,28,29]. The antisense strand of the siRNA serves as a template for the RNA-induced silencing complex (RISC) to recognize and cleave a complementary messenger RNA (mRNA), which is then rapidly degraded [30]. 

However, long dsRNAs evoke the interferon response in mammalian cells, leading to non-specific mRNA degradation and global inhibition of protein translation, rather than to specific gene silencing [31,32]. Therefore, siRNAs are used for RNAi-mediated gene silencing in mammalian cells [33]. 

Micro RNA (miRNA), another small RNA that induces gene silencing, was found to control development in *C. elegans* [34,35]. miRNAs are genome-encoded and transcribed as a single transcript that folds to form a stem-loop structure. These precursors are processed/cleaved by Drosha to leave double-stranded stem regions, which then mediate translational repression and/or degradation of target mRNAs [36,37]. This miRNA biogenesis machinery is also utilized to generate siRNAs from transgenic constructs [38,39]. 

dsRNA-mediated gene silencing is a novel technique for performing genome-wide screens in organisms in which forward genetic approaches are not practical. Since RNAi-mediated methods are theoretically applicable for almost all organisms in which a dsRNA delivery and/or expression system is available, the pertinent question is how dsRNA can be delivered to, or expressed in, cells and organisms.

## 3. Delivery and/or Expression of dsRNAs in Target Cells

*Drosophila* S2 cells and *C. elegans* readily incorporate long dsRNAs into their cells. For *Drosophila* S2 cells, dsRNAs are supplied in the culture medium and are directly incorporated into the cells, where they destroy target RNAs [10,11]. *C. elegans* can incorporate dsRNA by soaking in dsRNA solution [13,14] or feeding on *E. coli* expressing the relevant molecule [12,15]. Long dsRNAs are synthesized/expressed *in vitro* or in *E. coli* by bidirectional transcription from a DNA construct containing the coding region. 

As described in Section 2, in mammalian cells, siRNAs are used to avoid the interferon response triggered by long dsRNAs. siRNAs are chemically synthesized or enzymatically prepared [40], and then transfected into cells by conventional methods. However, their effects are transient in actively replicating cells, where the constant siRNA pool is continually diluted by cell division, which typically restricts the silencing effects to a period of less than two weeks. Consequently, repeated treatment with siRNA is necessary for sustained silencing; however, this sometimes seriously damages the cells. In addition, many mammalian cells are inaccessible to chemical or electrochemical methods of transfection. To overcome these limitations, another option, vector-mediated expression of hairpin type RNAs, was developed. Hairpin RNAs are transcribed from expression vectors as single stranded molecules that form a stem-loop structure. A loop connects the two complementary RNA fragments that create the double-stranded stem via base pairing. The dsRNAs in the stem regions are recognized and cleaved by Dicer and enter RISC as siRNAs. The vectors used for RNAi are based on viruses that infect mammalian cells, including adenovirus, adeno-associated virus, retroviruses and lentiviruses [41,42]. The length of the double-stranded stems is 19 to 29 bp in mammalian cells, whereas it is usually longer (~100 to 500 bp) in *Drosophila* due to the lack of interferon response.

Compared with S2 cells, it is difficult to deliver dsRNAs into the *Drosophila* organism. Early *Drosophila* RNAi-mediated gene silencing experiments involved manual injections of dsRNAs into *Drosophila* embryos [16,17]. Since manual injection is a labor-intense procedure requiring considerable technical expertise, no genome-wide screens have been performed using dsRNA-injection. To induce RNAi in *Drosophila*, libraries of fly strains bearing transgenes for expression of hairpin type RNAs have been established (Section 5). Since RNAi of essential genes is often lethal, regulation of hairpin RNA expression is required to maintain stable transgenic flies. In *Drosophila*, the Gal4/UAS (upstream activation sequence) system is commonly used to control gene expression [25]. Gal4/UAS dependent RNAi inducible strains are described in detail below (Section 5).

## 4. Genome-Wide RNAi in *Drosophila* Cultured Cells

There are several excellent reviews of cell-based genome-wide screens using RNAi [43,44,45]. However, we would like to briefly mention the unavoidable limitations of cell-based screens. Different cell lines express different sets of genes depending on their differentiation state and/or culture conditions, despite having the same genome. Therefore, the same screen can identify different subsets of genes depending on the cell lines used [46,47]. Another limit is the difficulty in addressing biological processes that cannot be recapitulated in cultured cells; for example, development, behavior, and immunity. If such biological phenomena could be reduced to simple cell-based processes, genome-wide screens using cultured cells would be worthwhile. For example, when the signal pathway important for a biological process of interest is known, genes controlling that pathway can be screened in cultured cells.

## 5. *Drosophila* RNAi Libraries

**Figure 1:**
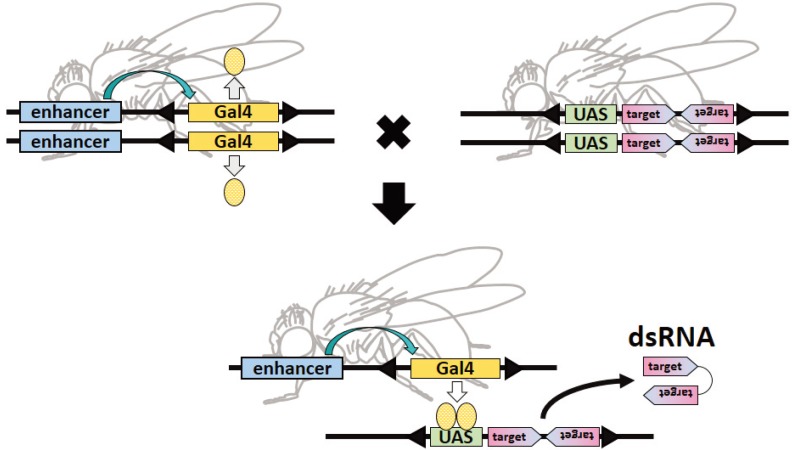
Inductive expression of double-stranded RNA (dsRNA) by the Gal4/UAS system. Fly strains expressing Gal4 proteins in a spatiotemporally regulated manner under the control of enhancers are genetically crossed with fly strains bearing a gene expressing hairpin RNA downstream of an UAS. In the progeny, dsRNA is expressed in a regulated pattern and induces its target gene silencing.

In contrast to *C. elegans*, *Drosophila* does not incorporate dsRNAs by feeding. In *Drosophila*, therefore, dsRNA is expressed in target cells as hairpin RNA (Section 3) using the Gal4/UAS system (Figure 1). The yeast Gal4 transcription factor binds to the UAS and activates expression of the downstream gene; theoretically, the gene downstream of the UAS is not expressed in the absence of Gal4. Consequently, a genetic cross between UAS- and Gal4-fly strains will induce expression of the gene downstream of the UAS. By placing genes expressing hairpin RNAs downstream of an UAS, RNAi is readily induced by genetic crossing. In addition, there are a large number of Gal4 strains in which the Gal4 gene is conditionally expressed, for example, in a specific tissue, or developmental stage, or under specific temperature conditions. Therefore, spatiotemporal patterns and levels of expression of hairpin RNAs are dependent on the Gal4 strain used.

### 5.1. Advantages

RNAi-based *in vivo* screening has advantages and disadvantages compared to conventional forward genetic methods. One of the advantages is the control that can be exercised over spatiotemporal gene silencing and knockdown efficiency by using an appropriate Gal4 strain. One large library of Gal4 strains is the NP collection, which was generated as a collection of enhancer trap strains, bearing an enhancerless Gal4 gene insertion [48]. The spatiotemporal expression patterns of these drivers are listed in the *Drosophila* Genetic Resource Center (DGRC) [49], which facilitates the selection of appropriate Gal4 drivers for screens with different aims. The knockdown efficiency can be controlled by selecting the Gal4 drivers with different expression levels. The drivers expressing different levels of Gal4 in a similar expression pattern provide variable knockdown efficiencies in the same tissues. In addition, UAS strains are another factor influencing knockdown efficiency. Since P-element mediated transformation results in the random insertion of transgenes into the *Drosophila* genome, different strains carry the same transgene frequently inserted into different genomic loci, which can lead to variable levels of transcriptional activity. Thus, knockdown efficiency can be regulated by selecting the appropriate transgenic strain. Temperature and the amount of Dicer are other factors that control knockdown efficiency. Higher temperatures (up to 28 °C), and/or increased amounts of Dicer, enhance knockdown efficiency through their influences on Gal4 activity and siRNA production, respectively.

Two major collections of *Drosophila* RNAi inducible strains have been generated independently by the National Institute of Genetics (NIG) [23] and the Vienna *Drosophila* RNAi center (VDRC) [22] (Table 1). These were essentially generated by the same methods but are distinct in many respects. Whereas the VDRC collection enables knockdown of 12,521 genes covering 88.2% of the *Drosophila* genome, the NIG collection enables the induced knockdown of 6,923 genes representing 48.8% of the genome. The lengths of dsRNAs in the VDRC and NIG collections are 300–400 bp and 500 bp, respectively. Since longer dsRNAs decrease target mRNA levels more efficiently than short ones, some genes, but not all, are more efficiently knocked down NIG strains than in VDRC strains [50]. As mentioned in the previous paragraph knockdown efficiency is also affected by where the UAS-hairpin interfering RNA (IR) transgene is inserted, as the location of the insertion site can affect hairpin RNA expression. Both VDRC and NIG collections provide more than one strain with the same gene inserted in a different location, which provides a choice of strains with different knockdown levels.

**Table 1:** *Drosophila* RNA interference (RNAi) libraries.

Institute/University	Vectors	Insertion sites	Reference
National Institute of Genetics	R57	random	[23]
Vienna Drosophila RNAi Center	GD, KK	random	[51]
Harvard Medical School	VALIUM	preinserted attP sites	[24]

### 5.2. Disadvantages

The disadvantages of RNAi-based *in vivo* screens are (1) erroneous results, such as false positives and negatives; and (2) variable gene silencing efficiency, depending on insertion sites; however, in some cases, variable gene silencing can be used advantageously, as described above. In some cases, false positives result from off-target effects (OTEs) caused by sequence similarities between dsRNAs and mRNAs, which leads to the silencing of untargeted genes. Since effective siRNA molecules are approximately 21 bases in length, of which a 19 base stretch is used for sequence matching, the sequence similarity of the 19 base fragments produced by long dsRNAs is a key factor in identifying off-target genes. In silico prediction of the OTEs of specific dsRNAs is provided on the dsCheck website [52] and the NIG and VDRC stock center websites ([23] and [53], respectively). Besides the original GD library, the VDRC collection also contains another set of RNAi strains, the KK library. dsRNAs in the KK library were designed to result in lower OTEs than the GD library. This is achieved by choosing target sequences that are highly specific to the targeted gene and that possess lower numbers of CAN repeats, which are common in the Drosophila genome [54].

The other disadvantage is that it is difficult to determine whether the lack of a phenotype associated with the knockdown of a specific locus is a genuine negative result or whether it is due to a low efficiency of gene knockdown. False negative results are thought to be mainly the result of low levels of dsRNA expression caused by positional effect of the insertion site of the UAS-IR transgene.

## 6. Genome-Wide *In Vivo* RNAi in *Drosophila*

*Drosophila in vivo* RNAi techniques have been used to screen both the whole genome and subsets of genes. Published *in vivo* screens are listed in Table 2. In addition, publications that employed the NIG RNAi library are summarized on the NIG website [55]. These screens are categorized into three classes. Class 1 encompasses screens that address questions that can only be addressed using *in vivo* studies, for example, questions about development, behavior, and immunity; Class 2 encompasses screens that address questions that can be answered by both *in vitro* and *in vivo* approaches; and Class 3 encompasses screens for genes that have been pre-identified by an *in vitro* screen and that require further validation by *in vivo* methods. In the following section, we briefly summarize *in vivo* screens that have been performed using *Drosophila* RNAi libraries.

**Table 2:** *In vivo* RNAi screens in *Drosophila*.

Class	Authors	Purpose: What kinds of genes are expected to be identified	Reference
Class 1	Cronin *et al*.	genes against intestinal infection with Serratia marcescens	[56]
	Osman *et al*.	suppressors of AML1-ETO	[57]
	Yamamoto-Hino *et al*.	genes involved in gylcosylation	[58]
	Avet-Rochex *et al*	melanotic tumor suppressor genes involved in blood cell homeostasis	[59]
	Neely *et al*.	genes involved in heart development and function	[60]
	Neely *et al*.	genes regulating pain	[61]
	Lesch *et al*.	genes required for wound closure	[62]
	Schnorrer *et al*.	genes involved in muscle morphogenesis and function	[63]
	Pospisilik *et al*.	genes involved in obesity	[64]
	Neumuller *et al*.	genes involved in stem cell differentiation	[65]
	Yano *et al*.	genes involved in apical transport	[66]
	Carney *et al*.	genes maintaining proper neuroblast numbers	[67]
	Valakh *et al*.	genes involved in formation, growth, and maintenance of the neuromuscular junction	[68]
Class 2	Mummery-Widmer *et al*.	Notch regulators	[69]
	Vosfeldt *et al*.	modifiers of polyQ dependent toxicity	[70]
	Llamusi *et al*.	modifiers of CTG repeat dependent toxicity	[71]
Class 3	Kambris *et al*.	serine protease genes required for Toll activation	[72]
	Saj *et al*.	Notch regulators	[73]
	Port *et al*.	genes involved in Wg secretion	[74]
	Du *et al*.	regulators of Hh pathway	[75]
	Aikin *et al*.	genes involved in Hh secretion	[76]

### 6.1. Class 1: Screens to Address Questions that Can only Be Investigated Using an *In Vivo* Approach

Cronin *et al*. assayed 10,689 different genes (78% of the *Drosophila* genome) that affect susceptibility to intestinal *Serratia marcescens* infection [56]. Of these, 8.3% (885 genes) were defined as hits; the majority (89.3%; 790 genes) were susceptibility candidates and 95 genes (10.7% of hits) were negative regulators. To determine whether the candidate genes functioned in the gut epithelium and/or in macrophage-like hemocytes, they performed tissue-specific knockdown experiments using different Gal4 drivers. Seventy-eight and 56 genes functioned were found to function only in the gut and hemocytes, respectively, and 79 functioned in both. Gene ontology (GO) enrichment analysis revealed a marked enhancement of genes associated with intracellular processes in the gut, such as endocytosis and exocytosis, proteolysis, vesicle-mediated transport, the stress response, immune system development, growth, stem cell division, and cell death. In hemocytes, genes involved in phagocytosis, including endocytosis, response to external stimuli, and vesicle trafficking, were enriched. They also performed analysis of the JAK-STAT pathway during *S. marcescens* infection and found that JAK-STAT signaling enhanced epithelial cell death, and positively regulated the compensatory proliferation of intestinal cells. In summary, more than 800 genes were identified, many of which were of unknown function, demonstrating that host defense may involve many processes not limited to the classical innate immune response pathways.

The t(8:21)(q22;q22) translocation, which produces the abnormal fusion protein AML1-ETO in humans, is associated with acute myeloid leukemia (AML). However, AML1-ETO alone is not sufficient to cause leukemia in mouse; secondary mutations are required before AML1-ETO-expressing cells become leukemogenic. To identify suppressors of AML1-ETO, Osman *et al*. chose to knockdown target genes in AML1-ETO-expressing cells using an *in vivo* RNAi strategy [57]. They screened UAS-IR transgenic lines, thereby targeting around 1,500 genes. Eight candidates were discovered. Among the candidates, they studied one gene, *Drosophila* calpainB, and mammalian calpains in detail, which revealed that they were required for AML1-ETO stabilization. This study suggests that *Drosophila* provides a promising, genetically tractable model to investigate the conserved basis of leukemogenesis.

Glycosylation has crucial regulatory roles in various biological processes. Yamamoto-Hino *et al*. performed a primary screened of 6923 UAS-IR strains for genes involved in the glycosylation of a neural glycoprotein and identified 171 candidates [58]. These were further validated by knockdown experiments, by using *in silico* analysis and a secondary set of UAS-IR strains that targeted regions distinct from those of the primary strains. To identify additional glycosylation genes, they performed searches for genes that interacted with those identified in the primary screen using the yeast two-hybrid database, BIOGRID, and the genetic interaction data listed in FlyBase. After validation experiments, a total of 109 genes were identified as glycosylation-related, 95 of which were newly assigned to this category. The gene functional groups obtained included glycosylation reactions, transcription, RNA regulation, translation, intracellular trafficking, cytoskeletal regulation, signal transduction, protein degradation, mitochondrial function, and other functions. Further analysis revealed that 17 of the identified genes were specific for *Drosophila* neural glycosylation. These data demonstrate that the use of interaction network databases and second target validation contributes to efficient screening and ultimately the production of conclusive results.

To identify melanotic tumor suppressor genes involved in blood cell homeostasis, Avet-Rochex *et al*. screened 1,341 genes (approximately 10% of all *Drosophila* genes) and validated the candidates obtained by re-screening independent secondary UAS-IR lines [59]. Finally, they identified 59 genes previously unlinked to blood cell development or function in *Drosophila*. The candidate genes were grouped into nine categories, namely protein synthesis, signaling, transcription, splicing, protein folding and stability, DNA replication/repair mitochondrial activity, membrane proteins targeting, and unknown function. The authors constructed an interaction network between the 59 genes using various databases (DroID, BioGrid, FlyBase), in addition to manual text-mining for each of the 59 genes and their mammalian or yeast orthologs. This approach allowed them to uncover several nodes of interaction among the genes, with 47 candidates linked to at least one other gene, suggesting that they function as complexes and/or in the same pathways.

Neely *et al*. screened 7,061 evolutionarily conserved genes for potential developmental and adult heart function defects [60]. They identified 498 loci that were classified into functional categories including signaling, ion transporter activity, metabolism and mitochondrial structure, development and morphogenesis, and transcriptional regulation. Among these, they confirmed that the CCR4-Not complex is involved in proper heart function in *Drosophila* and mice.

To identify novel genes regulating pain, Neely *et al*. tested 16,051 elav-Gal4 > UAS-IR combinations targeting 11,664 different *Drosophila* genes (82% of *Drosophila* genome) for their contribution to noxious temperature avoidance [61]. Positive hits were retested, and 622 specific transgenic UAS-IR strains, corresponding to 580 genes, were identified as candidate thermal nociception genes. Among these, the function of straightjacket, a member of the alpha2delta family of genes that function as subunits of voltage-gated calcium channels and control the function and development of synapses, was analyzed further. They also analyzed a mouse alpha2delta3 mutant model and showed that it also exhibited impaired behavioral heat pain sensitivity. These two mammalian studies by Neely *et al*. also indicated that *Drosophila in vivo* screens provide a promising system for investigation of regulatory systems conserved in metazoans.

Lesch *et al*. screened 142 genes to identify loci required for normal wound closure in *Drosophila* larval epidermis [62]. They identified two categories of candidate genes; the first comprised genes encoding components of stress-activated protein kinase (SAPK) signaling pathways, such as the canonical Jun kinase relay and associated transcription factors (11 genes in total), and the second comprised genes involved in actin cytoskeletal remodeling, including Rho-like GTPases and loci involved in phagocytosis (10 genes in total).

Schnorrer *et al*. screened 10,461 genes to find loci contributing to muscle morphogenesis and function [63]. Of these, targeting of 2,785 resulted in defects. The results of this study were systematically compared with those of previous studies to identify muscle genes that had either been identified by expression profiling or through chromatin immunoprecipitation of Mef2-binding sites in embryos. More than half of the muscle-expressed genes and almost half of the Mef2 targets were functionally validated in this screen. A total of 30 genes were positive in all three datasets and 13 of these had no functional assignment before this study.

Pospisilik *et al*. screened 10,489 genes using UAS-IR strains to identify candidate obesity genes, which resulted in the identification of 516 genes, 319 of which had human orthologs [64]. GO based pathway analysis for biological processes revealed enrichment of gene sets involved in cell fate determination, cellular protein metabolic processes, signal transduction, intracellular transport, and regulation of smoothened signaling. A network interaction assembly, based on the results of yeast-two-hybrid analysis, text-mining, and pathway database information analysis of *Drosophila* hits and their mammalian orthologs, revealed an interaction map that highlighted genes involved in development, nutrient transport, cell-cycle regulation, the proteasome, protein translation, and chromatin remodeling. The biological process “regulation of smoothened/Hedgehog signaling” was the top-scoring signal transduction pathway of all the annotated pathways in the primary screen. They further analyzed a fat-specific Sufu knockout mouse (Sufu is a potent endogenous inhibitor of Hedgehog signaling in mammals) and found that Hedgehog activation blocks white but not brown adipocyte differentiation in the mammalian model system. This mammalian study also revealed a signal conserved in metazoan obesity.

To identify genes that control the balance between neural stem cell (neuroblast) self-renewal and differentiation, Neumuller *et al*. screened 12,314 individual genes (VDRC GD) by examining whether knockdown of each caused abnormalities in number, size or shape, or intracellular GFP fused to CD8 (CD8-GFP) accumulation of neuroblasts, ganglion mother cells, or intermediate neural progenitors [65]. The quality of the result was evaluated by re-screening of a subset of the candidates using a second RNAi library, the KK library (described in Section 5). The authors concluded that the reproducibility of the candidate genes is 78.5%. The candidate genes were clustered using databases containing data obtained from two-hybrid screens, biochemical analysis, interlog, text-mining, and genetic interactions between *Drosophila* genes. Among the candidates, in addition to ribosome subunit genes, genes involved in splicing control and transcriptional elongation and chromatin remodeling were found to have important roles in neuroblast self-renewal and differentiation.

The distinct localization of membrane proteins with regard to cell polarity is crucial for the structure and function of various organs in multicellular organisms. To identify genes involved in the regulation of protein localization, Yano *et al*. performed a large-scale screen using a *Drosophila* RNAi library [66]. *Drosophila* photoreceptor cells have a morphologically distinct apico-basal polarity, along which Chaoptin (Chp), a glycosylphosphatidylinositol (GPI)-anchored membrane protein, and Na/K ATPase are localized to the apical and basolateral domains, respectively. By examining the subcellular localization of these proteins, they identified 106 genes whose knockdown resulted in their mislocalization. GO analysis revealed that the knockdown of proteasome components resulted in mislocalization of Chp to the basolateral plasma membrane, suggesting the direct or indirect involvement of the proteasome in the selective localization of Chp to the apical plasma membrane of *Drosophila* photoreceptor cells.

To find regulators of neural progenitor self-renewal, Carney *et al*. first performed microarray analysis to identify genes expressed in neuroblasts [67]. They further selected 595 genes that had available UAS-IR strains and mammalian orthologs. By performing a neuroblast-specific, RNAi-based functional screen, 84 genes were identified to be required for proper maintenance of neuroblast numbers. These genes are excellent candidates for regulating neural progenitor self-renewal in *Drosophila* and probably also in mammals.

Valakh *et al*. knocked down 2,970 genes by neuron-specific RNAi in a search for genes involved in the formation, growth, and maintenance of the neuromuscular junction (NMJ) [68]. Knockdown of 158 genes in post-mitotic neurons led to abnormalities in the neuromuscular system. Bioinformatics analysis demonstrated that genes with overlapping annotated functions were enriched within the hits for each phenotype, suggesting shared biological roles are important for this aspect of synaptic development. For example, genes for proteasome subunits and mitotic spindle organizers were enriched among those whose knockdown led to defects in synaptic apposition and NMJ stability. Their findings highlight the potential importance of proteasome function for active zone development and maintenance in the presynaptic compartment.

### 6.2. Class 2: Screens to Identify New Genes that Can Be Found by both *In Vivo* and *In Vitro* Experiments

Mummery-Widmer *et al*. screened 11,619 genes to find Notch regulators and identified six new loci involved in asymmetric cell division and 23 involved in the regulation of the Notch signaling pathway [69]. Further analysis of protein interaction data revealed that nuclear import pathways and the COP9 signallosome are important for Notch regulation.

A large-scale RNAi screen in *Drosophila* was performed to identify modifiers of the toxicity induced by expression of truncated Ataxin-3, which results in polyQ expression diseases. Vosfeldt *et al*. screened 6,930 genes for which a human ortholog could be identified in a *Drosophila* RNAi library [70]. When the resulting candidate genes were overlaid onto the meta-interaction network produced by Costello *et al*. [77], a set of proteasomal proteins previously implicated in polyQ toxicity were found to be involved. The authors compared their data with those from P/EP-element-based screens for the polyQ-induced rough eye phenotype [78], and RNAi screens for modifiers of polyQ aggregation, performed in cultured insect cells [79] and *C. elegans* [80]. However, a small overlap of candidates was found among them.

Among 1,215 UAS-IR strains, Llamusi *et al*. isolated 202 lines that showed modification of an eye phenotype induced by expression of CTG repeats [71]. To exclude eye-specific suppressors of CTG toxicity, the 202 lines were investigated for a wing phenotype also caused by expression of CTG repeats, which resulted in the identification of 34 modifiers.

### 6.3. Class 3: Screens to Find True Positives among Candidate Genes Identified in *In Vitro* Screens

Infection of *Drosophila* with pathogens is thought to lead to processing of the ligand of the Toll receptor, Spaetzle (Spz), by secreted serine proteases (SPs). However, knowledge of SPs acting upstream of Spz in regulating the Toll pathway is scarce. Kambris *et al*. screened 75 distinct *Drosophila* SP genes and identified five novel SPs [72], including the Spz processing enzyme (SPE), which directly cleaves Spz [81].

Saj *et al*. performed a cell-based RNAi screen of 14,200 genes (BKN library) [82] and identified 900 candidate Notch regulators [73]. Subsequently, they used a *Drosophila* RNAi library for *in vivo* validation of the candidates in wing and eye development and confirmed 333 of 501 tested genes as Notch regulators. By mapping the phenotypic attributes of their data onto an interaction network, they identified another 68 relevant genes and found several modules of unexpected Notch regulatory activity. A total of 401 Notch regulators were compared to two other data sets of Notch regulators; one was obtained by studying genetic interactions and mutant phenotypes (Flybase) [83] and the other was obtained from a whole genome *in vivo* RNAi screen focused on genes involved in external sensory organ formation. There was a very limited overlap between the three data sets, which most likely reflected the differences in the approaches used to generate them.

Port *et al*. performed a genome-wide RNAi screen to identify genes involved in the secretion of Wingless (Wg) [74]. For the primary screen, *Drosophila* S2R+ cell lines that stably expressed a Wg-Renilla luciferase (WgRluc) fusion protein or a secreted Renilla luciferase (sRluc) were established to assay specific defects in Wg secretion. A library of dsRNAs targeting more than 14,000 *Drosophila* genes was screened in the generated S2R+ cell lines and 387 were assigned as primary hits. Following pre-exclusion of genes with well-documented functions unrelated to protein secretion, 115 were re-screened for eye and wing phenotypes using a *Drosophila* RNAi library. A p24 protein, Éclair, and a protein termed Sorting nexin 3 (Snx3) were identified as hits in more than one assay.

To identify novel regulators of the Hedgehog (Hh) pathway among genes functioning in the UPS, Du *et al*. first selected 248 UPS genes from the *Drosophila* genome [75]. UAS-IR strains targeting 238 UPS genes were screened by examining adult wing blade phenotypes, the distribution patterns of full length Cubitus interruptus (CiFL), the transcription factors of the Hh pathway, and the expression of the dpp gene, a direct transcriptional target of Hh signaling, in the wing disc. Among these 238 genes, two novel loci (dUba3 and dUuc12) were found to be negative regulators of Hh signaling activity.

Aikin *et al*. screened ~21,000 dsRNAs using S2 cells transfected with the Hh gene fused to Renilla luciferase (Hh-Ren) in a search for regulators of cholesterol-modified Hedgehog secretion [76]. This identified 125 genes, which were then evaluated by secondary dsRNAs that did not overlap with those used in the primary screen. This led to the high confidence identification of 24 genes whose depletion significantly affected Hh-Ren secretion; 11 had previously been found in an RNAi screen for regulators of general protein secretion, while the others had no previously known role in secretion. Four genes (CG5964, CG8441, CG3305, and CG12693) were further analyzed using *Drosophila* UAS-IR strains.

The various applications of RNAi libraries mentioned above indicate that they are a useful and straightforward system for performing genetic screens and *in vivo* validation. In addition, mammalian studies of genes identified in *Drosophila* (by Osman, Pospisilik and Neely) exemplify the power of *Drosophila in vivo* RNAi screens for the discovery of pivotal pathways conserved among metazoa.

## 7. Erroneous Results and Possible Solutions

As demonstrated by the studies of Saj *et al*. [73] and others [46,47,70], the results of similar screens may reveal a low level of overlap in the genes that are identified. This suggests that these large-scale RNAi screens lead to high numbers of both false positive and false negative results, although in some cases this may be due to differences in the approaches used for screening.

One potential source of false positives in RNAi-based screens comes from OTEs that occur when a dsRNA has homology to mRNAs that are not the intended target. Minimization of sequence similarities between dsRNAs and non-target mRNAs decreases the number of false positives; however, it does not entirely eliminate the problem. Thus, candidate gene validation is required following primary screens. There are two ways to evaluate candidate genes [84]. One approach involves using multiple dsRNAs for each gene identified as a hit in a primary screen. This is based on the fact that multiple dsRNAs homologous to different regions of a gene, but not to each other, are unlikely to affect the same non-target mRNAs; therefore, if more than one unrelated dsRNA targeting the same mRNA exhibits the same phenotype, it is likely that this is a genuine consequence of knockdown of the intended target mRNA, rather than an off-target effect. The other approach involves rescuing the dsRNA-induced phenotype by expression of a functional version of the target gene that is resistant to the dsRNA. Such transcripts, homologous to the target gene at the amino acid level but not at the nucleotide sequence level, may be found in other species. For these cross-species rescue experiments, a simple method to generate constructs has been developed [85]. Genomic clones for 11 different *Drosophila* species are now publicly available. These clones contain a unique loxP site that can accept a new DNA cassette via Cre/loxP-mediated recombination. Since the retrofitting vector for transgenesis also contains a loxP site, the genomic clones and retrofitting vector can be easily fused by Cre activity to generate rescue constructs for the generation of transgenic *Drosophila* strains. 

False negatives in large-scale RNAi-based screens are mainly caused by the inefficient knockdown of specific target genes by particular RNAi reagents under the conditions used. A meta-analysis of several genome-wide, cell-based *Drosophila* RNAi screens suggested that the rate of false negative results is at least 8% [86]. To decrease false negative rates, three validation protocols have been proposed. The first approach is to decrease positional effect of the insertion site of the UAS-IR transgene, which sometimes causes low levels of dsRNA expression. PhiC integrase-mediated transformation, based on site-specific recombination between the attB and attP recognition sites, was developed to obtain transgenic strains in which transgenes are inserted into defined loci containing the attP sequence; these sites have been termed “landing sites” [87,88,89,90]. Unlike P-element based vectors, the phiC-mediated transformation vector contains the attB site, which allows integration at pre-integrated attP sites in the *Drosophila* genome. Responsiveness to Gal4 was measured in strains with UAS inserted at different landing sites [91,92]. Some landing sites inducing strong expression can be used to generate UAS-IR strains with fewer false negative results. Based on this information, a new RNAi library using the VALIUM vector [39] is being generated in a collaboration between NIG and Harvard University [24,93] (Table 1). The second approach is to use multiple, independently screened dsRNAs per gene [86]. In principle, the use of multiple dsRNAs per gene should reduce the number of false negatives, as a single ineffective dsRNA would be compensated for by those that are effective. However, since simply increasing the number of dsRNAs will also result in higher false positive rates, careful consideration must be given to the disambiguation of inconsistent results obtained with multiple dsRNAs directed against the same target gene. The third approach is to re-screen a subset of genes enriched for potential positives [94,95]; this is similar to the approach proposed for generating comprehensive interactome maps. The information that is now available in gene and protein interaction maps has the potential to serve as a guide for identifying such subsets of genes. Protein interaction network data can be used to guide re-screening efforts to generate more comprehensive and accurate lists of genes involved in specific biological processes.

## 8. *In Vivo* RNAi in Mice

Mouse (*Mus musculus*) is a widely used mammalian model organism. Although mice can be bred easily in laboratories, it is difficult to handle the large number of strains (greater than 20,000) required for genome-wide screens. However, deliberate screening strategies and the use of pools of shRNAs targeting some or all genes in the mouse genome has enabled screening to be performed with less than 100 mice (Table 3). Tumorigenesis has been studied extensively using *in vivo* RNAi screens [96,97,98]. In these experiments, pools of vectors expressing shRNAs targeting approximately 300 to 1,000 selected genes were transfected into hepatocytes or hematopoietic stem cells from tumor model mice, which were then transplanted into recipient mice. After tumors were formed from the transplanted cells, the shRNA-expressing vectors were recovered and shRNAs that affected tumor development were identified by determining those that were under or overrepresented compared to an untransfected pool of shRNA-expressing vectors. Enriched and depleted shRNAs were then subjected to further validation experiments and functional analyses.

**Table 3:** *In vivo* RNAi screens in mice.

Authors	Purpose: what kinds of genes are expected to be identified	Number of shRNAs	Reference
Zender *et al*.	genes involved in hepatocarcinogenesis	631	[96]
Bric *et al*.	genes involved in lymphomagenesis	2,300	[97]
Maecham *et al*.	genes involved in lymphoma prgression	2,250	[98]
Wuestefeld *et al*.	genes involved in liver regeneration	631	[99]
Vaeble *et al*.	genes involved in viral replication	>10,000	[100]
Beronja *et al*.	genes involved in epidermal growth	>77,000	[101]

Interestingly, Maecham *et al*. performed an *in vitro* RNAi screen by culturing transfected lymphoma cells in culture dishes in parallel with an *in vivo* screen by injecting the lymphoma cells to mice and found that the set of shRNAs that had effects on lymphoma cell proliferation *in vitro* was largely different to the set that had effects on palpable lymphoma development *in vivo* [98]. This result suggests that an *in vivo* screen is necessary to identify sets of genes that affect tumor growth under physiologically relevant conditions.

Direct transfection of transposable elements expressing shRNAs was used to search for genes involved in liver regeneration [99]. Pools of 631 selected shRNAs were introduced into mouse liver cells and then the livers were damaged by carbon tetrachloride treatment. Genomic DNA was isolated from dissected regenerated livers and deep sequencing was performed to quantify shRNA abundance. The enriched shRNAs were then validated and analyzed further.

Recently, a large-scale screen and a genome-wide screen were performed to identify mouse genes involved in viral replication [100] and embryonic epidermal growth [101], respectively. For the viral replication study, Varble *et al*. generated libraries of viruses expressing more than 10,000 shRNAs and examined their replication abilities in mice. For the epidermal cell growth study, Beronja *et al*. transfected a library of viruses expressing more than 77,000 shRNAs, which targeted 15,991 mouse genes, into embryonic epidermis and then identified enriched and depleted shRNAs by comparing their abundances in the initial pools and the pools collected from epidermal cells. In both cases, differentially expressed shRNAs were validated and analyzed further.

Although large-scale *in vivo* RNAi screens were developed recently, these methods depend on the detection of changes in shRNA abundance; therefore, a large-scale screen cannot be applied to the study of more complex biological processes, such as development and behavior. However, improvement of screening strategies will enable the application of *in vivo* RNAi screens to investigations of various biological events in mammals.

## 9. Conclusions

Since RNAi was discovered, the phenomenon has been applied to various biological fields as a tool for gene silencing. In the beginning, this was mainly performed in cultured cells and *C. elegans*. When RNAi was combined with the Gal4/UAS system in *Drosophila*, it allowed RNAi to be induced in a spatiotemporal manner. Using this inducible RNAi technique, large-scale screens for various biological processes have been performed successfully in *Drosophila*, proving that this RNAi-based *in vivo* screen is straightforward and valuable. However, screening results to date have revealed that RNAi-based screens have relatively high levels of false positives and negatives. To disambiguate the screening results, experimental and computational analyses have been proposed. Such improvements will increase the accuracy of RNAi-based screen results.

## References

[1] Fire A., Xu S., Montgomery M.K., Kostas S.A., Driver S.E., Mello C.C. (1998). Potent and specific genetic interference by double-stranded RNA in Caenorhabditis elegans. Nature.

[2] *C. elegans* Sequencing Consortium (1998). Genome sequence of the nematode *C. elegans*: A platform for investigating biology. Science.

[3] Adams M.D., Celniker S.E., Holt R.A., Evans C.A., Gocayne J.D., Amanatides P.G., Scherer S.E., Li P.W., Hoskins R.A., Galle R.F. (2000). The genome sequence of *Drosophila melanogaster*. Science.

[4] Lander E.S., Linton L.M., Birren B., Nusbaum C., Zody M.C., Baldwin J., Devon K., Dewar K., Doyle M., FitzHugh W. (2001). Initial sequencing and analysis of the human genome. Nature.

[5] Venter J.C., Adams M.D., Myers E.W., Li P.W., Mural R.J., Sutton G.G., Smith H.O., Yandell M., Evans C.A., Holt R.A. (2001). The sequence of the human genome. Science.

[6] Meister G., Tuschl T. (2004). Mechanisms of gene silencing by double-stranded RNA. Nature.

[7] Silva J., Chang K., Hannon G.J., Rivas F.V. (2004). RNA-interference-based functional genomics in mammalian cells: Reverse genetics coming of age. Oncogene.

[8] Ghildiyal M., Zamore P.D. (2009). Small silencing RNAs: An expanding universe. Nat. Rev. Genet..

[9] Whitehurst A.W., Bodemann B.O., Cardenas J., Ferguson D., Girard L., Peyton M., Minna J.D., Michnoff C., Hao W., Roth M.G. (2007). Synthetic lethal screen identification of chemosensitizer loci in cancer cells. Nature.

[10] Boutros M., Kiger A.A., Armknecht S., Kerr K., Hild M., Koch B., Haas S.A., Paro R., Perrimon N. (2004). Genome-wide RNAi analysis of growth and viability in *Drosophila* cells. Science.

[11] Ulvila J., Parikka M., Kleino A., Sormunen R., Ezekowitz R.A., Kocks C., Ramet M. (2006). Double-stranded RNA is internalized by scavenger receptor-mediated endocytosis in *Drosophila* S2 cells. J. Biol. Chem..

[12] Timmons L., Fire A. (1998). Specific interference by ingested dsRNA. Nature.

[13] Tabara H., Grishok A., Mello C.C. (1998). RNAi in *C. elegans*: Soaking in the genome sequence. Science.

[14] Maeda I., Kohara Y., Yamamoto M., Sugimoto A. (2001). Large-scale analysis of gene function in Caenorhabditis elegans by high-throughput RNAi. Curr. Biol..

[15] Lehner B., Tischler J., Fraser A.G. (2006). RNAi screens in Caenorhabditis elegans in a 96-well liquid format and their application to the systematic identification of genetic interactions. Nat. Protoc..

[16] Kennerdell J.R., Carthew R.W. (1998). Use of dsRNA-mediated genetic interference to demonstrate that frizzled and frizzled 2 act in the wingless pathway. Cell.

[17] Misquitta L., Paterson B.M. (1999). Targeted disruption of gene function in *Drosophila* by RNA interference (RNA-i): A role for nautilus in embryonic somatic muscle formation. Proc. Natl. Acad. Sci. USA.

[18] Lam G., Thummel C.S. (2000). Inducible expression of double-stranded RNA directs specific genetic interference in *Drosophila*. Curr. Biol..

[19] Fortier E., Belote J.M. (2000). Temperature-dependent gene silencing by an expressed inverted repeat in *Drosophila*. Genesis.

[20] Martinek S., Young M.W. (2000). Specific genetic interference with behavioral rhythms in *Drosophila* by expression of inverted repeats. Genetics.

[21] Giordano E., Rendina R., Peluso I., Furia M. (2002). RNAi triggered by symmetrically transcribed transgenes in *Drosophila melanogaster*. Genetics.

[22] Dietzl G., Chen D., Schnorrer F., Su K.C., Barinova Y., Fellner M., Gasser B., Kinsey K., Oppel S., Scheiblauer S. (2007). A genome-wide transgenic RNAi library for conditional gene inactivation in *Drosophila*. Nature.

[23] NIG-FLY. http://www.shigen.nig.ac.jp/fly/nigfly/about/aboutRnai.jsp/.

[24] Transgenic RNAi Project. http://www.flyrnai.org/TRiP-HOME.html/.

[25] Brand A.H., Perrimon N. (1993). Targeted gene expression as a means of altering cell fates and generating dominant phenotypes. Development.

[26] Zamore P.D., Tuschl T., Sharp P.A., Bartel D.P. (2000). RNAi: Double-stranded RNA directs the ATP-dependent cleavage of mRNA at 21 to 23 nucleotide intervals. Cell.

[27] Elbashir S.M., Lendeckel W., Tuschl T. (2001). RNA interference is mediated by 21- and 22-nucleotide RNAs. Genes Dev..

[28] Elbashir S.M., Harborth J., Lendeckel W., Yalcin A., Weber K., Tuschl T. (2001). Duplexes of 21-nucleotide RNAs mediate RNA interference in cultured mammalian cells. Nature.

[29] Bernstein E., Caudy A.A., Hammond S.M., Hannon G.J. (2001). Role for a bidentate ribonuclease in the initiation step of RNA interference. Nature.

[30] Hammond S.M., Bernstein E., Beach D., Hannon G.J. (2000). An RNA-directed nuclease mediates post-transcriptional gene silencing in *Drosophila* cells. Nature.

[31] Karpala A.J., Doran T.J., Bean A.G. (2005). Immune responses to dsRNA: Implications for gene silencing technologies. Immunol. Cell Biol..

[32] Gantier M.P., Williams B.R. (2007). The response of mammalian cells to double-stranded RNA. Cytokine Growth Factor Rev..

[33] Bantounas I., Phylactou L.A., Uney J.B. (2004). RNA interference and the use of small interfering RNA to study gene function in mammalian systems. J. Mol. Endocrinol..

[34] Lee R.C., Feinbaum R.L., Ambros V. (1993). The *C. elegans* heterochronic gene lin-4 encodes small RNAs with antisense complementarity to lin-14. Cell.

[35] Wightman B., Ha I., Ruvkun G. (1993). Posttranscriptional regulation of the heterochronic gene lin-14 by lin-4 mediates temporal pattern formation in *C. elegans*. Cell.

[36] Lee Y., Ahn C., Han J., Choi H., Kim J., Yim J., Lee J., Provost P., Radmark O., Kim S. (2003). The nuclear RNase III Drosha initiates microRNA processing. Nature.

[37] Yates L.A., Norbury C.J., Gilbert R.J. (2013). The long and short of microRNA. Cell.

[38] Chang K., Elledge S.J., Hannon G.J. (2006). Lessons from Nature: MicroRNA-based shRNA libraries. Nat. Methods.

[39] Ni J.Q., Zhou R., Czech B., Liu L.P., Holderbaum L., Yang-Zhou D., Shim H.S., Tao R., Handler D., Karpowicz P. (2011). A genome-scale shRNA resource for transgenic RNAi in *Drosophila*. Nat. Methods.

[40] Sandy P., Ventura A., Jacks T. (2005). Mammalian RNAi: A practical guide. Biotechniques.

[41] Couto L.B., High K.A. (2010). Viral vector-mediated RNA interference. Curr. Opin. Pharmacol..

[42] Pan Q., van der Laan L.J., Janssen H.L., Peppelenbosch M.P. (2012). A dynamic perspective of RNAi library development. Trends Biotechnol..

[43] Mohr S., Bakal C., Perrimon N. (2010). Genomic screening with RNAi: Results and challenges. Annu. Rev. Biochem..

[44] Flockhart I.T., Booker M., Hu Y., McElvany B., Gilly Q., Mathey-Prevot B., Perrimon N., Mohr S.E. (2012). FlyRNAi.org—The database of the *Drosophila* RNAi screening center: 2012 update. Nucleic Acids Res..

[45] Schmidt E.E., Pelz O., Buhlmann S., Kerr G., Horn T., Boutros M. (2013). GenomeRNAi: A database for cell-based and *in vivo* RNAi phenotypes, 2013 update. Nucleic Acids Res..

[46] Liu T., Sims D., Baum B. (2009). Parallel RNAi screens across different cell lines identify generic and cell type-specific regulators of actin organization and cell morphology. Genome Biol..

[47] Sepp K.J., Hong P., Lizarraga S.B., Liu J.S., Mejia L.A., Walsh C.A., Perrimon N. (2008). Identification of neural outgrowth genes using genome-wide RNAi. PLoS Genet..

[48] Hayashi S., Ito K., Sado Y., Taniguchi M., Akimoto A., Takeuchi H., Aigaki T., Matsuzaki F., Nakagoshi H., Tanimura T. (2002). GETDB, a database compiling expression patterns and molecular locations of a collection of Gal4 enhancer traps. Genesis.

[49] Drosophila Genetic Resource Center. http://www.dgrc.kit.ac.jp/.

[50] Yamamoto-Hino M., Goto S. (2013).

[51] Vinna *Drosophila* RNAi Center. http://stockcenter.vdrc.at/control/main/.

[52] dsCheck. http://dscheck.rnai.jp/.

[53] Vinna *Drosophila* RNAi Center Definition. http://stockcenter.vdrc.at/control/vdrcdefinition/.

[54] Meadows L. (2013). Personal communication.

[55] Research Resource Circulation. http://rrc.nbrp.jp/index.jsp/.

[56] Cronin S.J., Nehme N.T., Limmer S., Liegeois S., Pospisilik J.A., Schramek D., Leibbrandt A., Simoes Rde M., Gruber S., Puc U. (2009). Genome-wide RNAi screen identifies genes involved in intestinal pathogenic bacterial infection. Science.

[57] Osman D., Gobert V., Ponthan F., Heidenreich O., Haenlin M., Waltzer L. (2009). A *Drosophila* model identifies calpains as modulators of the human leukemogenic fusion protein AML1-ETO. Proc. Natl. Acad. Sci. USA.

[58] Yamamoto-Hino M., Kanie Y., Awano W., Aoki-Kinoshita K.F., Yano H., Nishihara S., Okano H., Ueda R., Kanie O., Goto S. (2010). Identification of genes required for neural-specific glycosylation using functional genomics. PLoS Genet..

[59] Avet-Rochex A., Boyer K., Polesello C., Gobert V., Osman D., Roch F., Auge B., Zanet J., Haenlin M., Waltzer L. (2010). An *in vivo* RNA interference screen identifies gene networks controlling *Drosophila melanogaster* blood cell homeostasis. BMC Dev. Biol..

[60] Neely G.G., Kuba K., Cammarato A., Isobe K., Amann S., Zhang L., Murata M., Elmen L., Gupta V., Arora S. (2010). A global *in vivo Drosophila* RNAi screen identifies NOT3 as a conserved regulator of heart function. Cell.

[61] Neely G.G., Hess A., Costigan M., Keene A.C., Goulas S., Langeslag M., Griffin R.S., Belfer I., Dai F., Smith S.B. (2010). A genome-wide *Drosophila* screen for heat nociception identifies alpha2delta3 as an evolutionarily conserved pain gene. Cell.

[62] Lesch C., Jo J., Wu Y., Fish G.S., Galko M.J. (2010). A targeted UAS-RNAi screen in *Drosophila* larvae identifies wound closure genes regulating distinct cellular processes. Genetics.

[63] Schnorrer F., Schonbauer C., Langer C.C., Dietzl G., Novatchkova M., Schernhuber K., Fellner M., Azaryan A., Radolf M., Stark A. (2010). Systematic genetic analysis of muscle morphogenesis and function in *Drosophila*. Nature.

[64] Pospisilik J.A., Schramek D., Schnidar H., Cronin S.J., Nehme N.T., Zhang X., Knauf C., Cani P.D., Aumayr K., Todoric J. (2010). *Drosophila* genome-wide obesity screen reveals hedgehog as a determinant of brown versus white adipose cell fate. Cell.

[65] Neumuller R.A., Richter C., Fischer A., Novatchkova M., Neumuller K.G., Knoblich J.A. (2011). Genome-wide analysis of self-renewal in *Drosophila* neural stem cells by transgenic RNAi. Cell Stem Cell.

[66] Yano H., Yamamoto-Hino M., Awano W., Aoki-Kinoshita K.F., Tsuda-Sakurai K., Okano H., Goto S. (2012). Identification of proteasome components required for apical localization of Chaoptin using functional genomics. J. Neurogenet..

[67] Carney T.D., Miller M.R., Robinson K.J., Bayraktar O.A., Osterhout J.A., Doe C.Q. (2012). Functional genomics identifies neural stem cell sub-type expression profiles and genes regulating neuroblast homeostasis. Dev. Biol..

[68] Valakh V., Naylor S.A., Berns D.S., DiAntonio A. (2012). A large-scale RNAi screen identifies functional classes of genes shaping synaptic development and maintenance. Dev. Biol..

[69] Mummery-Widmer J.L., Yamazaki M., Stoeger T., Novatchkova M., Bhalerao S., Chen D., Dietzl G., Dickson B.J., Knoblich J.A. (2009). Genome-wide analysis of Notch signalling in *Drosophila* by transgenic RNAi. Nature.

[70] Vo S.H., Butzlaff M., Pru S.K., Ni Charthaigh R.A., Karsten P., Lankes A., Hamm S., Simons M., Adryan B., Schulz J.B. (2012). Large-scale screen for modifiers of ataxin-3-derived polyglutamine-induced toxicity in *Drosophila*. PLoS One.

[71] Llamusi B., Bargiela A., Fernandez-Costa J.M., Garcia-Lopez A., Klima R., Feiguin F., Artero R. (2013). Muscleblind, BSF and TBPH are mislocalized in the muscle sarcomere of a *Drosophila* myotonic dystrophy model. Dis. Model. Mech..

[72] Kambris Z., Brun S., Jang I.H., Nam H.J., Romeo Y., Takahashi K., Lee W.J., Ueda R., Lemaitre B. (2006). *Drosophila* immunity: A large-scale *in vivo* RNAi screen identifies five serine proteases required for Toll activation. Curr. Biol..

[73] Saj A., Arziman Z., Stempfle D., van Belle W., Sauder U., Horn T., Durrenberger M., Paro R., Boutros M., Merdes G. (2010). A combined *ex vivo* and *in vivo* RNAi screen for notch regulators in *Drosophila* reveals an extensive notch interaction network. Dev. Cell..

[74] Port F., Hausmann G., Basler K. (2011). A genome-wide RNA interference screen uncovers two p24 proteins as regulators of Wingless secretion. EMBO Rep..

[75] Du J., Zhang J., Su Y., Liu M., Ospina J.K., Yang S., Zhu A.J. (2011). *In vivo* RNAi screen reveals neddylation genes as novel regulators of Hedgehog signaling. PLoS One.

[76] Aikin R., Cervantes A., D'Angelo G., Ruel L., Lacas-Gervais S., Schaub S., Therond P. (2012). A genome-wide RNAi screen identifies regulators of cholesterol-modified hedgehog secretion in *Drosophila*. PLoS One.

[77] Costello J.C., Dalkilic M.M., Beason S.M., Gehlhausen J.R., Patwardhan R., Middha S., Eads B.D., Andrews J.R. (2009). Gene networks in *Drosophila melanogaster*: Integrating experimental data to predict gene function. Genome Biol..

[78] Bilen J., Bonini N.M. (2007). Genome-wide screen for modifiers of ataxin-3 neurodegeneration in *Drosophila*. PLoS Genet..

[79] Zhang S., Binari R., Zhou R., Perrimon N. (2010). A genomewide RNA interference screen for modifiers of aggregates formation by mutant Huntingtin in *Drosophila*. Genetics.

[80] Nollen E.A., Garcia S.M., van Haaften G., Kim S., Chavez A., Morimoto R.I., Plasterk R.H. (2004). Genome-wide RNA interference screen identifies previously undescribed regulators of polyglutamine aggregation. Proc. Natl. Acad. Sci. USA.

[81] Jang I.H., Chosa N., Kim S.H., Nam H.J., Lemaitre B., Ochiai M., Kambris Z., Brun S., Hashimoto C., Ashida M. (2006). A Spatzle-processing enzyme required for toll signaling activation in *Drosophila* innate immunity. Dev. Cell.

[82] Horn T., Arziman Z., Berger J., Boutros M. (2007). GenomeRNAi: A database for cell-based RNAi phenotypes. Nucleic Acids Res..

[83] Flybase. http://flybase.org/.

[84] Echeverri C.J., Beachy P.A., Baum B., Boutros M., Buchholz F., Chanda S.K., Downward J., Ellenberg J., Fraser A.G., Hacohen N. (2006). Minimizing the risk of reporting false positives in large-scale RNAi screens. Nat. Methods.

[85] Kondo S., Booker M., Perrimon N. (2009). Cross-species RNAi rescue platform in *Drosophila melanogaster*. Genetics.

[86] Booker M., Samsonova A.A., Kwon Y., Flockhart I., Mohr S.E., Perrimon N. (2011). False negative rates in *Drosophila* cell-based RNAi screens: A case study. BMC Genomics.

[87] Groth A.C., Fish M., Nusse R., Calos M.P. (2004). Construction of transgenic *Drosophila* by using the site-specific integrase from phage phiC31. Genetics.

[88] Bateman J.R., Lee A.M., Wu C.T. (2006). Site-specific transformation of *Drosophila* via phiC31 integrase-mediated cassette exchange. Genetics.

[89] Venken K.J., He Y., Hoskins R.A., Bellen H.J. (2006). P[acman]: A BAC transgenic platform for targeted insertion of large DNA fragments in *D. melanogaster*. Science.

[90] Bischof J., Maeda R.K., Hediger M., Karch F., Basler K. (2007). An optimized transgenesis system for *Drosophila* using germ-line-specific phiC31 integrases. Proc. Natl. Acad. Sci. USA.

[91] Markstein M., Pitsouli C., Villalta C., Celniker S.E., Perrimon N. (2008). Exploiting position effects and the gypsy retrovirus insulator to engineer precisely expressed transgenes. Nat. Genet..

[92] Pfeiffer B.D., Ngo T.T., Hibbard K.L., Murphy C., Jenett A., Truman J.W., Rubin G.M. (2010). Refinement of tools for targeted gene expression in *Drosophila*. Genetics.

[93] Kondo S., Ueda R. (2013). Personal communication.

[94] Wang L., Tu Z., Sun F. (2009). A network-based integrative approach to prioritize reliable hits from multiple genome-wide RNAi screens in *Drosophila*. BMC Genomics.

[95] Guest S.T., Yu J., Liu D., Hines J.A., Kashat M.A., Finley R.L. (2011). A protein network-guided screen for cell cycle regulators in *Drosophila*. BMC Syst. Biol..

[96] Zender L., Xue W., Zuber J., Semighini C.P., Krasnitz A., Ma B., Zender P., Kubicka S., Luk J.M., Schirmacher P. (2008). An oncogenomics-based *in vivo* RNAi screen identifies tumor suppressors in liver cancer. Cell.

[97] Bric A., Miething C., Bialucha C.U., Scuoppo C., Zender L., Krasnitz A., Xuan Z., Zuber J., Wigler M., Hicks J. (2009). Functional identification of tumor-suppressor genes through an *in vivo* RNA interference screen in a mouse lymphoma model. Cancer Cell.

[98] Meacham C.E., Ho E.E., Dubrovsky E., Gertler F.B., Hemann M.T. (2009). *In vivo* RNAi screening identifies regulators of actin dynamics as key determinants of lymphoma progression. Nat. Genet..

[99] Wuestefeld T., Pesic M., Rudalska R., Dauch D., Longerich T., Kang T.W., Yevsa T., Heinzmann F., Hoenicke L., Hohmeyer A. (2013). A Direct *in vivo* RNAi screen identifies MKK4 as a key regulator of liver regeneration. Cell.

[100] Varble A., Benitez A.A., Schmid S., Sachs D., Shim J.V., Rodriguez-Barrueco R., Panis M., Crumiller M., Silva J.M., Sachidanandam R. (2013). An *in vivo* RNAi screening approach to identify host determinants of virus replication. Cell Host Microbe.

[101] Beronja S., Janki P., Heller E., Lien W.H., Keyes B.E., Oshimori N., Fuchs E. (2013). RNAi screens in mice identify physiological regulators of oncogenic growth. Nature.

